# Differences in Spatio-Temporal Behavior of Zebrafish in the Open Tank Paradigm after a Short-Period Confinement into Dark and Bright Environments

**DOI:** 10.1371/journal.pone.0019397

**Published:** 2011-05-02

**Authors:** Denis B. Rosemberg, Eduardo P. Rico, Ben Hur M. Mussulini, Ângelo L. Piato, Maria E. Calcagnotto, Carla D. Bonan, Renato D. Dias, Rachel E. Blaser, Diogo O. Souza, Diogo L. de Oliveira

**Affiliations:** 1 Departamento de Bioquímica, Universidade Federal do Rio Grande do Sul, Porto Alegre, Rio Grande do Sul, Brazil; 2 Instituto Nacional de Ciência e Tecnologia em Excitotoxicidade e Neuroproteção (INCT-EN), Porto Alegre, Rio Grande do Sul, Brazil; 3 Departamento de Biologia Celular e Molecular, Pontifícia Universidade Católica do Rio Grande do Sul, Porto Alegre, Rio Grande do Sul, Brazil; 4 Instituto Nacional Translacional em Medicina (INCT-TM), Porto Alegre, Rio Grande do Sul, Brazil; 5 Department of Psychology, University of San Diego, San Diego, California, United States; University of Chicago, United States of America

## Abstract

The open tank paradigm, also known as novel tank diving test, is a protocol used to evaluate the zebrafish behavior. Several characteristics have been described for this species, including scototaxis, which is the natural preference for dark environments in detriment of bright ones. However, there is no evidence regarding the influence of “natural stimuli” in zebrafish subjected to novelty-based paradigms. In this report, we evaluated the spatio-temporal exploratory activity of the short-fin zebrafish phenotype in the open tank after a short-period confinement into dark/bright environments. A total of 44 animals were individually confined during a 10-min single session into one of three environments: black-painted, white-painted, and transparent cylinders (dark, bright, and transparent groups). Fish were further subjected to the novel tank test and their exploratory profile was recorded during a 15-min trial. The results demonstrated that zebrafish increased their vertical exploratory activity during the first 6-min, where the bright group spent more time and travelled a higher distance in the top area. Interestingly, all behavioral parameters measured for the dark group were similar to the transparent one. These data were confirmed by automated analysis of track and occupancy plots and also demonstrated that zebrafish display a classical homebase formation in the bottom area of the tank. A detailed spatio-temporal study of zebrafish exploratory behavior and the construction of representative ethograms showed that the experimental groups presented significant differences in the first 3-min vs. last 3-min of test. Although the main factors involved in these behavioral responses still remain ambiguous and require further investigation, the current report describes an alternative methodological approach for assessing the zebrafish behavior after a forced exposure to different environments. Additionally, the analysis of ethologically-relevant patterns across time could be a potential phenotyping tool to evaluate the zebrafish exploratory profile in the open tank task.

## Introduction

The open field is the most used test for animal psychology studies in basic sciences. It consists basically of introducing an animal into a plain arena to observe its behavior across a specific range of time [1]. This test, usually performed with experimental rats or mice, provides an index of general behavior [2], [3], and in particular, exploratory activity, which is a crucial response to novelty [4]–[6]. The initial responses to the open field test of adult rats consist in thigmotaxis and increased exploratory activity, which substantially decreases during the trial, reflecting an intra-session state of habituation [7]–[9]. The intra-session habituation involves spatial working memory and may also represent deeper neurobiological constructs, such as adaptive processing of sensory information and development of a cognitive map [10]–[12]. Furthermore, animals tend to establish during the test a key location (homebase), characterized as a “safe” place to which they repeatedly return after exploring the environment and spend more time during the trial [13], [14]. Thus, the open field test offers a valuable and reliable test of activity and sequential (spatio-temporal) structure of the exploratory behavior [3], [15], which emerges as an interesting tool that reveals the animal's interaction with a novel environment [16], [17].

Zebrafish is becoming a popular animal model for behavioral neuroscience studies [18]–[20]. Although the use of zebrafish in behavioral research is increasing rapidly, the full potential offered by its use in these studies still needs further elucidation. Similar to the open field used for rodents, the novel tank diving test – also known as open tank paradigm – is emerging as a task for behavioral analysis in zebrafish. This test fundamentally consists in evaluate its vertical exploratory activity based on the tendency of this species to initially dive to the bottom and gradually swim to upper areas of the tank [18]. Several reports have been undertaken in order to characterize the zebrafish responses to novelty-based paradigms [16], [21]–[24]. Recent data showed that zebrafish display a robust habituation response to novelty [12] and the establishment of a homebase [25]. Furthermore, pharmacological studies have demonstrated that anxiogenic and anxiolytic drugs can influence the habituation response to the novel tank test and induce changes in some endpoint behaviors, such as freezing, erratic movements, hyperactivity, and bottom-dwelling (or diving) [12], [16], [19], [21]–[24], [26], [27]. However, due to the complexity of the behavioral repertoire displayed by adult zebrafish, the behaviors themselves still remain poorly understood [28].

A straightforward approach in the validation of behavioral measures for this species was performed using the bright/dark apparatus [28]–[31]. This task is characterized by the natural preference of zebrafish for dark environments (scototaxis), an innate feature previously suggested for its usefulness for the development of behavioral paradigms [32]. In fact, the bright/dark test allowed the organization of ethograms, which show relevant dimensions of defensive behavior [29], [31]. This advance was taken for the first time by Blaser et al. [28], using the dark/bright tank in zebrafish. The confinement to each environment demonstrated that animals with a high avoidance of the bright side displayed substantial amount of freezing behavior when forcefully exposed to the bright chamber. Moreover, a recent study using a different protocol also demonstrated that animals forcefully exposed to the white compartment three consecutive times presented substantial differences in the behavioral repertoire observed in the light/dark tank [29]. Although these reports strongly suggest the aversion of zebrafish to bright environments, there is no data evaluating the effect of “natural stimuli” on the zebrafish behavior in the novel tank. Thus, it may be equally interesting to determine how the forced short-period exposure in two distinct preferred environments (dark vs. bright) affect their spatio-temporal exploratory activity [28], [29], [32].

Therefore, the aim of the current study was to investigate the spatio-temporal exploratory activity of the short-fin zebrafish phenotype in the novel tank test after a short-period confinement into dark and bright environments. The purpose to map the behavioral repertoire typically employed by the species in the open tank task lead us to suggest a standard exploratory profile for the confined groups.

## Methods

### Ethics statement

All procedures with animal subjects have been approved by the Ethics Committee for Use of Animals – CEUA from Universidade Federal do Rio Grande do Sul (protocol number 2008058).

### Animals

Adult male and female zebrafish (*Danio rerio*) (4–6 months-old, ∼50∶50 male∶female ratio) of heterogeneous wild-type stock (standard short-fin phenotype) were obtained from a local commercial supplier (Delphis, RS, Brazil). Fish were housed in 50-L aquariums (80–100 fish per aquarium) for at least 2 weeks prior to the experiments in order to acclimate to the animal facility. All tanks were filled with unchlorinated water previously treated with 132 µL.L^−1^ AquaSafe® (Tetra, VA, USA) and kept under mechanical and chemical filtration at a targeted temperature of 26±2°C and water pH at 7.0–8.0. The room illumination was provided by ceiling-mounted fluorescent light tubes on a 12/12 light/dark photoperiod cycle (lights on at 7:00 am). Animals were fed twice a day until satiety with a commercial flake fish food (alcon BASIC®, Alcon, Brazil). All animals used in this study were experimentally naive, healthy and free of any signs of disease. They were maintained according to the National Institute of Health Guide for Care and Use of Laboratory Animals.

### Apparatuses and experimental procedures

**Figure 1:**
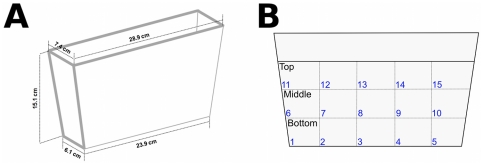
The novel tank. (A) The apparatus consisted in a trapezoidal plastic tank with the specific dimensions described above. (B) Virtual divisions were used for evaluation of zebrafish swimming activity in the novel tank diving test, with three vertical areas (bottom, middle, and top) and fifteen horizontal sections (1–15), with five sections per area.

The behavioral test was performed during the same time frame each day (between 10:00 am and 4:00 pm). All apparatuses were filled with water adjusted to home tanks conditions and the experimental procedures were performed in a stable surface with all environmental distractions kept to a minimum. A total of 44 animals obtained in five separate batches were used for independent behavioral experiments. The forced exposures to the different environments were performed during a 10-min period. Animals were randomically handled from their home tanks and individually transferred to the confinement cylinder (7.5 cm diameter×12.5 cm high) filled with 0.5 L of aquarium-treated water. Fish from both sexes (∼50∶50 male∶female ratio) were used for each experimental group. For the bright confinement (white group, *n* = 16), fish were placed in a white-painted cylinder, whereas the dark confinement (black group, *n* = 16) was performed in a black-painted cylinder. Another group of fish was confined in a transparent cylinder (transparent group, *n* = 12), which closely resembled the home tanks. After the forced exposure period, animals were carefully removed from their respective confinement cylinder and placed in the novel tank where their behavioral activity was recorded. This apparatus consisted in a trapezoidal plastic tank (23.9 cm along the bottom×28.9 cm at the top×15.1 cm high and 15.9 cm along the diagonal side. It was 7.4 cm wide at the top, and tapered to 6.1 cm at the bottom) (**Figure 1A**) filled with 1.5 L of aquarium treated water. The dimension of the apparatus was similar to those previously described for the zebrafish novel tank test [12], [16], [21]–[24]. A webcam (Microsoft® LifeCam 1.1 with Auto-Focus) was placed 40 cm from the testing tank to ensure that the apparatus was within the camera vision range and it was used to monitor the location and swimming activity of the fish. Two yellow sheets of paper (standard letter size: 21.59 cm×27.94 cm) were placed 4.3 cm behind the tank to ensure a uniform background for the video analysis. In order to boost the contrast between the background and zebrafish, two 60-watts light bulbs were placed 40 cm behind the yellow screen. The webcam was plugged to a computer to record and analyze the videos using appropriate automated video-tracking software.

The trapezoidal tank was virtually divided into three equally horizontal areas (bottom, middle, and top) in order to evaluate vertical exploratory activity. To analyze the horizontal exploratory activity, the tank was also virtually divided into fifteen sections, with five sections per area as demonstrated in **Figure 1B**. Once the animals were placed in the novel test tank, the recording was started. Each subject was observed individually in a single session and the behavior was recorded over a period of 15 min. Before and after the test, oxygen levels in water of the apparatuses were measured and remained adequate during the experiment (8 ppm, Labcom Test®, SC, Brazil).

### Behavioral analysis

The behavioral analysis was performed in a laptop computer using ANY-maze® software (Stoelting CO, USA) to track the swimming activity of the animals at a rate of 30 frames/sec. The video-tracking data were then used to determine relevant measures of vertical exploration across time, such as time spent per area and transitions to each area. Moreover, some endpoint behaviors were measured during the test, including distance travelled, absolute turn angle, meandering, average speed, and time mobile. The absolute turn angle represents the sum of all vectors angle of movements created from one position to animal's center point to the next. The anti-clockwise movement was considered negative and clockwise movement positive (−180° to 180°C). From this measure we calculated the meandering, which is the result of the absolute turn angle divided by the total distance travelled. In addition to the time spent, number of transitions, and the latency to middle and top area transitions, the vertical exploratory activity was assessed by measuring, in each area, the distance travelled, absolute turn angle, and meandering. The evaluation of the horizontal exploratory activity of zebrafish was performed by determining the time spent in each section per area and the number of transitions between sections per area. The ratio between the number of transitions per section and number of transitions per area was calculated to estimate the exploratory profile of fish considering both horizontal and vertical parameters (≤1 values predominantly characterize vertical exploration in each section, whereas >1 values indicate the predominance of horizontal exploration in the respective section). The distribution of the animals during the novel tank test was also evaluated by representative tracks, occupancy plots, and 3D reconstruction graphs. To establish a general profile of the exploratory activity, we created representative ethograms from each confined group by analyzing the 6-min behavioral responses. These ethograms were analyzed more specifically by comparing the first 3-min vs. last 3-min of test, which allowed a detailed evaluation of the exploratory activity of zebrafish during the intra-session habituation period [12].

### 3D Track reconstruction across time

The spatio-temporal analysis of zebrafish behavior in the novel tank diving test was also performed using track reconstruction across time as described previously [16], [24]. Briefly, the videos were analyzed using the ANY-maze® software with the coordinates of the experimental tank properly calibrated. The track data for each fish was exported as raw data into separate spreadsheets, providing spatial coordinates (x center and y center) across a time scale broken down into fractions of a second. The exported traces were analyzed based on similarity to each other by two trained observers (inter-rater reliability >0.85), on a consensus basis. The middle trace was selected as representative for the group, to illustrate the pattern of exploration (first 3-min vs. last 3-min of test). Spatio-temporal 3D reconstructions were created with Graphis 3D graphing software® in which the x center (horizontal distribution), y center (vertical distribution), and time were plotted on the X-, Z- and Y-axis, respectively.

### Statistics

Data were expressed as mean ± standard error of the mean (S.E.M.) and *p*-values were considered significant for *p*≤0.05. All behavioral parameters evaluated across time were analyzed by repeated-measures analysis of variance (ANOVA). The endpoint behavioral measures for vertical and horizontal exploration, homebase parameters, and the exploratory profile (transitions ratio) were analyzed by two-way ANOVA. Comparison among means was carried out using Bonferroni's test as post hoc. The basic data of general locomotor activity (distance travelled, average speed, absolute turn angle, meandering, and time mobile) and the comparison of homebase parameters between the experimental groups were analyzed by one-way ANOVA, followed by Bonferroni's test as post hoc.

## Results

### Vertical exploration

**Figure 2:**
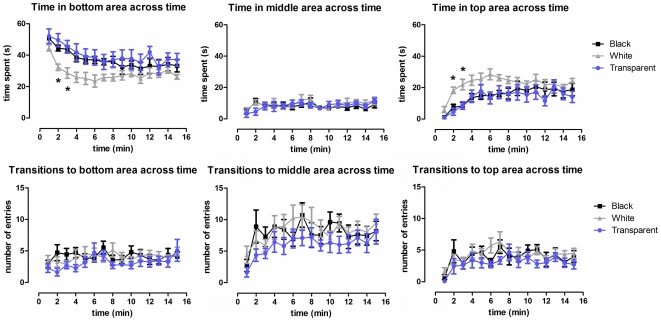
Vertical exploration of zebrafish previously confined into dark, bright, and transparent environments. The exploratory activity in each vertical area (bottom, middle, and top) was assessed during a 15-min trial and the time spent and number of transitions per area were shown. * Significant difference between black/transparent and white cylinder-confined groups (repeated-measures ANOVA followed by Bonferroni's test as post hoc, *p*≤0.05).

In the 15-min novel tank test (**Figure 2**), the black, white, and transparent cylinder-confined groups showed a characteristic pattern of duration in the different areas (bottom, middle and top) and in transitions between these vertical areas across time. A 3×15 (Color×Time) repeated-measures ANOVA was used to analyze the duration in each of the three vertical areas. We observed that the duration in the bottom decreased across the 15-min test (*F* [14,660] = 9.18, *p*<0.0001) and both black and transparent-confined groups spent significantly more time in the bottom than the white-confined group (*F* [2,660] = 7.33, *p*<0.005). Moreover, the time spent in the bottom area dropped faster in the white-confined group than in the other experimental groups (*F* [28,660] = 2.12, *p*<0.05). Although there was no significant effects of any variable on time in the middle area, the duration in the top increased significantly across the 15-min test (*F* [14,660] = 10.23, *p*<0.0001). Both black and transparent-confined groups spent significantly less time in the top than the white-confined group (*F* [2,660] = 5.83, *p*<0.01). Finally, the time spent in the top increased faster in the white-confined group than in the black and transparent-confined groups (*F* [28,660] = 2.12, *p*<0.05). Regarding the number of transitions between the three vertical areas, the animals displayed few entries to the middle and top areas (*F* [14,660] = 4.25, *p*<0.0001; and *F* [14,660] = 4.12, *p*<0.0001; respectively) in the first minute when compared to subsequent minutes of test.

### Horizontal exploration

**Figure 3:**
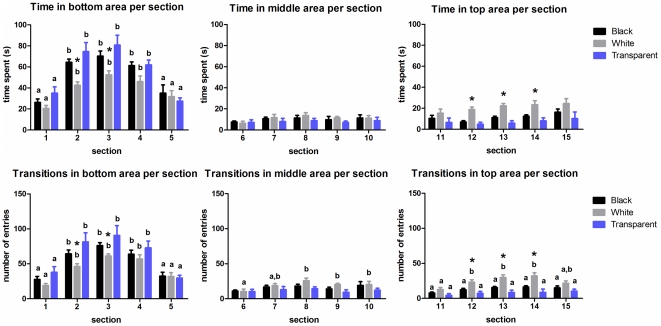
Effect of the confinement in the horizontal exploratory activity of zebrafish in the open tank. * Significant difference between black/transparent and white cylinder-confined groups. Distinct letters mean statistically significant differences within groups (two-way ANOVA followed by Bonferroni's test as post hoc, *p*≤0.05).

Since the time spent in bottom and top areas for animals previously exposed to dark, bright, and transparent environments reached a plateau after 7 min, further behaviors were assessed using the initial 6-min period. The horizontal exploratory activity was analyzed by two-way ANOVA using the duration of time spent and the transitions between each horizontal section (**Figure 3**). In general, animals spent more time in central sections of the bottom area (2, 3 and 4), while this preference for the center was less evident in the middle and top areas. Additionally, both black and transparent-confined fish spent significantly more time in the central sections of the bottom area than did white-confined animals, while the white-confined fish spent significantly more time in the central sections of the top area. The same pattern of results was observed for transitions between sections in each area.

**Figure 4:**
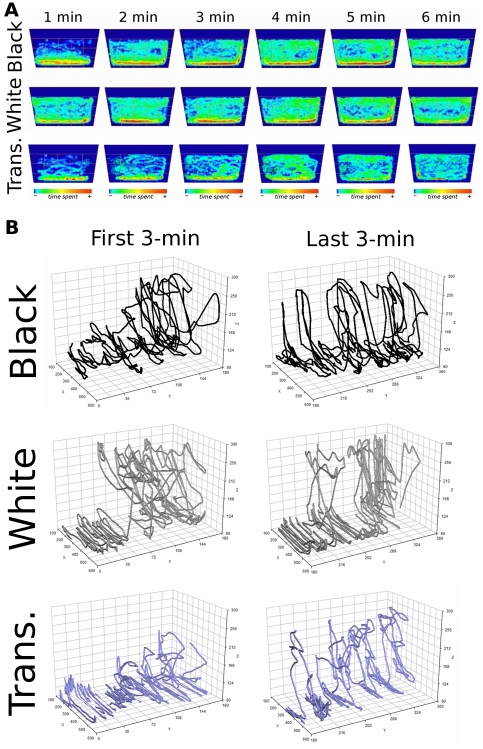
Comparison of the spatio-temporal behavior of the experimental groups in the novel tank test. (A) Representative occupancy plots of black, white, and transparent-confined groups displaying the specific patterns of time spent in each segment of the apparatus across time. Data were analyzed using video-tracking software (ANY-maze®, Stoelting CO, USA). (B) Representative 3D reconstructions of zebrafish swimming activity during the first 3-min vs. last 3-min of test obtained by plotting animal traces across the time. The X-, Z- and Y-axis represent the horizontal distribution, vertical distribution, and time, respectively.

Representative occupancy plots across time were constructed (**Figure 4A**), as well as a detailed 3D reconstruction of behavior (**Figure 4B**), which illustrate the differences between the three groups in terms of both lateral and vertical exploration (**see video S1**).

### Endpoint behaviors

**Figure 5:**
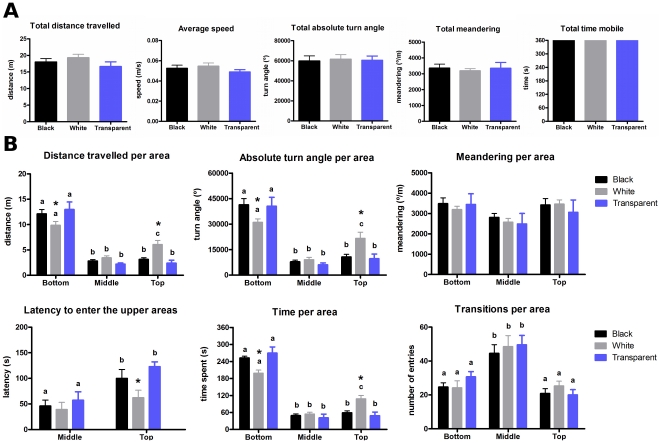
Basic endpoint behaviors of the experimental groups in the novel tank test during 6 min. (A) The graph shows total distance travelled, average speed, absolute turn angle, meandering, and time mobile. Data were analyzed by one-way ANOVA followed by Bonferroni's test as post hoc, considering *p*≤0.05 as significant. (B) Endpoint parameters of zebrafish behavior filtered by each vertical area (bottom, middle, and top) of the novel tank. * Significant difference between black/transparent and white cylinder-confined groups. Distinct letters mean statistically significant differences within groups (two-way ANOVA followed by Bonferroni's test as post hoc, *p*≤0.05).

The general basic behaviors, such as total distance travelled, absolute turn angle, meandering, and average speed did not significantly differ between the experimental groups (**Figure 5A**). It is interesting to mention that animals did not freeze at all during the novel tank test; they explored the apparatus during the entire 15-min trial and travelled a constant distance across time.

These endpoint behaviors were then filtered by vertical location and analyzed using two-way ANOVA (**Figure 5B**). Total swim distance, absolute turn angle, and duration in each area showed identical patterns of results: they were significantly higher in the bottom than in the middle or top areas. Additionally, they were significantly higher in the bottom area for black and transparent-confined animals, and significantly higher in the top area for white-confined animals. Meandering and transition frequency did not change between groups. However, latency to enter in the top was significantly shorter in the white-confined group than in the black and transparent-confined groups.

### Homebase formation

**Figure 6:**
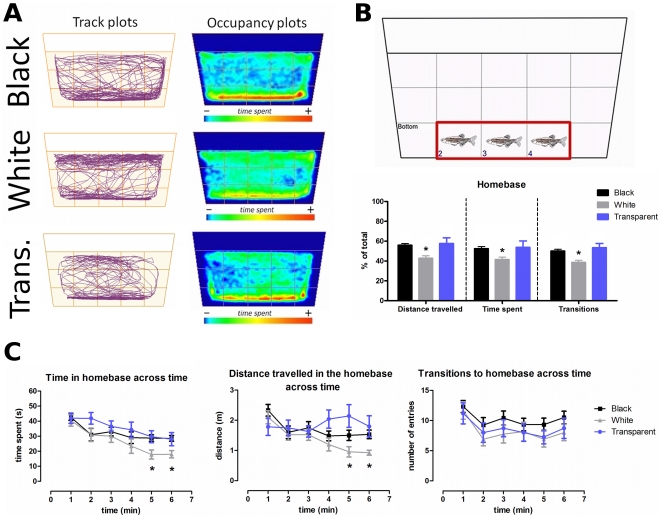
Overall exploratory activity and homebase formation of the experimental groups in the open tank. (A) Representative track and occupancy plots of the experimental groups obtained by video-tracking software (ANY-maze®, Stoelting CO, USA), displaying the specific patterns of their exploratory behavior during 6 min. (B) Zebrafish displays a classical homebase formation in the central sections of bottom area during the 6-min trial. Basic endpoint behaviors in the homebase were compared for black, white, and transparent cylinder-confined animals. * Significant difference between black/transparent and white cylinder-confined groups (one-way ANOVA followed by Bonferroni's test as post hoc, *p*≤0.05). (C) Evaluation of behavioral parameters of zebrafish in the homebase across time. * Significant difference between black/transparent and white cylinder-confined groups (repeated-measures ANOVA followed by Bonferroni's test as post hoc, *p*≤0.05).

Representative endpoint data (**Figure 6A**) illustrate the differences in the swimming traces among the areas and sections and also show that all groups spent significantly more time in the bottom area than the middle or top. These data allowed us to identify a classical homebase formation for the short-fin zebrafish strain in the novel tank test (middle sections of the bottom area). In this place, the animals travelled a greater distance, spent the most part of the test, and also performed a considerable number of entries. Fish confined into the white cylinder exhibited a significant decrease in all homebase parameters as compared to the groups forcefully exposed to the black and transparent cylinders (**Figure 6B**).

The analysis of the homebase parameters across time by a 3×6 (Color×Time) repeated-measures ANOVA showed that the duration and distance travelled in the homebase decreased across the 6-min test (*F* [5,264] = 11.25, *p*<0.0001 and *F* [5,264] = 4.66, *p*<0.001), respectively. The confinement into distinct environments also promoted significant differences in the time spent (*F* [2,264] = 4.35, *p*<0.05) and distance travelled in the homebase (*F* [2,264] = 4.90, *p*<0.05), which were significantly lower for the white cylinder-confined group during the 5th and 6th minutes of test. However, no significant differences in the number of homebase transitions between groups (**Figure 6C**) were observed. In the 15-min test, all homebase parameters remained similar to those observed in the final of the 6-min analysis (data not shown).

### Spatio-temporal patterns of behavior

**Figure 7:**
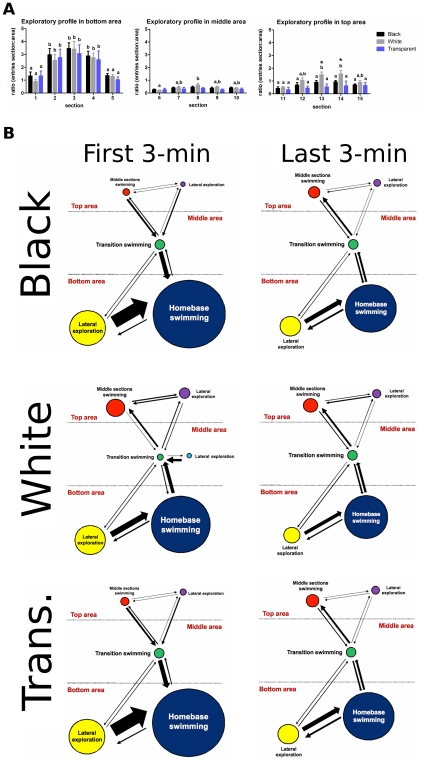
Behavioral profile of zebrafish in the open tank after the environmental manipulations. (A) The exploratory profile of dark, bright, and transparent-confined groups was determined by the ratio between the total transitions between sections and the number of entries in the respective area. * Significant difference between black/transparent and white cylinder-confined groups. Distinct letters mean statistically significant differences within groups (two-way ANOVA followed by Bonferroni's test as post hoc, *p*≤0.05). (B) Distinct behavior patterns displayed by zebrafish in the novel tank task after the short-period confinement into different environments. The ethological profiles were constructed by specifically analyzing the exploratory behaviors presented during the intra-session habituation period to the open tank. Representative ethograms were generated based on frequencies and transitions between each individual behavioral activity. The diameter of each circle corresponds to the frequency of each individual behavioral activity, whereas the arrow width and direction reflect the frequency of transitions between these behaviors.

The relative exploratory activity across both dimensions of the novel tank was estimated by calculating the ratio of transitions between horizontal sections to transitions between vertical areas (**Figure 7A**). These ratios, analyzed across time (**Figure S1**), were then used to create representative visual diagrams (ethograms) that reflect frequencies and transitions between each individual behavioral activity [24], [33]–[35] and to characterize the overall spatio-temporal exploratory pattern during the trial. Ethograms for the black, white, and transparent cylinder-confined groups were generated for the novel tank test during the first 3-min and last 3-min of the test (**Figure 7B**). The diameter of each circle corresponded to the frequency of each individual behavioral activity, while the arrow width and direction reflected the frequency of transitions between these behaviors. This ethological analysis allowed us to define the differences in the main behaviors presented by the experimental groups during the intra-session habituation period to the open tank, such as homebase swimming, lateral exploration, and transition swimming between bottom and top areas (**see video S1**).

## Discussion

The main finding of this study is that a short-period confinement into dark and bright environments induces differences in the spatio-temporal structure of zebrafish behavior in the open tank paradigm. Previous studies demonstrated the usefulness of the novel tank test to evaluate the vertical exploration of fish after exposure to several drugs [21], [23], [24], [27]. However, since adult zebrafish has been consolidated as an emergent vertebrate model in behavioral neuroscience research [28]–[31], it becomes reasonable to evaluate the effect promoted by “natural stimuli” in the behavioral repertoire of fish subjected to the open tank paradigm. The protocol consisted in confining the animals into a black or a white cylinder (dark vs. bright environments) during 10 min prior to the novel tank test. Additionally, another group of fish was confined into a transparent cylinder (transparent environment), which closely resembled the home tanks. Our results showed that all groups steadily increased their vertical exploratory activity within the first 6-min of the test, reaching a plateau after the 7th minute. These data corroborate with previous findings which demonstrated a rapid habituation response of zebrafish in the novel tank test [12], [23], [36]. Our results support the hypothesis that the behavioral manifestation of habituation responses to novelty in zebrafish is different from that of rodents [12]. Instead of a reduced locomotion when rodents become familiar with the novel environment [37], [38], zebrafish appears to do the opposite. Furthermore, motor and posture patterns that are known to be exhibited in the open tank trial, such as freezing and erratic movements [12], [16], [18], [19], were absent during our test. Studies demonstrated that both behaviors may significantly decrease over the habituation course to the novel tank [12], [24], or even occur with an extremely low frequency during the trial [18], [19], [39]. It is likely that several factors can explain these discrepancies, including differences in testing apparatuses (e.g. light intensity for the tracking) and in the protocol used (e.g. isolating the fish before the behavioral test). Thus, our data suggest that the time spent in the top and the number of transitions to top area across time may be better behavioral indicators of habituation response to the open tank.

Although we observed a rapid habituation response to novel tank test in all experimental groups, the confinement into bright environments altered the intra-session habituation response. Such as many teleosts, zebrafish displays a natural preference for dark environments in opposition to brightly lit ones, named scototaxis [29], [40]. Studies have been suggested that it represents a typical defensive pattern of species that exploits crypsis with the substratum as a strategy for predator avoidance [31], [40]. This task has already been validated at construct level for zebrafish [29]–[31] and recent pharmacological data also give a robust support for its predictive validity [41]. Additionally, Lau et al. [42] demonstrated that fish that highly avoided a bright image presented a significant activation of the medial zone of the dorsal telencephalic region (Dm) and the dorsal nucleus of the ventral telencephalic area (Vd), which is anatomically homolog to the mammalian amygdala and striatum, respectively. It has been shown that the scototaxis test did not present intra- or inter-session habituation of white avoidance [29], even though the authors could not reliably record the vertical distribution of zebrafish in the apparatus due to technical difficulties (e.g. in this task recordings must be made from top). Since zebrafish display a natural preference for dark environments, it is interesting that animals confined to the white cylinder habituate to the novel tank more rapidly than those confined to the black and the transparent cylinders. On the assumption that the white chamber is aversive, the obvious prediction is that white-confined fish should habituate less readily to the novel tank – a prediction which is inconsistent with our data. The faster habituation of the white-confined group is difficult to interpret, and highlights the need for a clearer understanding of the interaction between motivational state and vertical exploratory behavior in zebrafish. In the open tank trial, the total distance travelled, absolute turn angle, meandering, average speed, and time mobile did not change between the experimental groups, which indicate that the general locomotor activity of fish remained unaltered after the forced exposure to distinct environments. To better understand the nature of this effect, a more detailed evaluation of the spatio-temporal exploratory behavior across the intra-session habituation period was undertaken.

The sub-division of the novel tank in different sections allowed the estimation of the exploratory profile of the dark, bright, and transparent groups by considering the exploration of fish in both dimensions of the apparatus. In all three groups, fish show significantly more horizontal (lateral) exploratory activity in the central sections of bottom, whereas the middle area was mainly used for vertical transitions, in which animals practically did not explore. However, the top area ratio suggests that white-confined fish showed more lateral exploration in the upper portion of the tank than black and transparent-confined fish. These data were confirmed by representative track and occupancy plots, and suggest a characteristic homebase formation in the open tank paradigm by all three groups. The homebase is defined as a place in the field for which the experimental animal shows a preference across time, both in terms of occupancy and as a starting and ending point of exploratory excursions [14]. It has been shown that similarly to rodent behavior, zebrafish display a typical homebase formation in novelty-based paradigms [25]. Our 6-min observation period demonstrated that the white-confined group differed significantly on all homebase parameters assessed. The analysis of homebase behavior across time demonstrated that even though the transitions to the homebase remained virtually unaltered, fish confined into the white cylinder travelled a shorter distance and spent less time in the homebase during the 5th and 6th minutes. These data show that animals confined into the bright environment transit more rapidly out of homebase behaviors than those confined into the dark and transparent environments. It is possible that, additionally to being a reference point for the exploratory incursions, the homebase reflects a behavioral state comparable to thigmotaxis, and that confinement to the white cylinder disrupted this behavior. In this regard, Maximino et al. [29] showed that confining animals thrice in the white compartment prior to the scototaxis experiment does not alter spatio-temporal measures of preference, but decrease the frequency of burst swimming, freezing and thigmotaxis in the white compartment, suggesting that this treatment could diminish fear.

Our apparent inconsistency between predicted aversion to the white, and subsequent exploratory behavior in the novel tank may also support at dissociation between the mechanisms of black/white preference and novel tank diving behavior [43]. For example, the novel tank seems to be sensitive to diazepam, but not to chlordiazepoxide, while the scototaxis test is sensitive to other benzodiazepines as well [41]. Both behavioral paradigms also present different sensitivities to fluoxetine [23], [41] and the light/dark tank shows a lack of sensitivity for moclobemide, a MAO-A inhibitor [41]. These apparently conflicting data provided by pharmacological manipulations suggest that the two paradigms may not assess the same underlying state. Although the current study provides a detailed account of zebrafish behavioral repertoire in the open tank, further experimentations using alternative methodological approaches will be required to understand how these behaviors relate to that observed in the black/white preference task, and the neural mechanisms involved in each.

The spatio-temporal 3D reconstructions across the intra-session habituation period (first 3-min vs. last 3-min of test) showed that the white-confined fish displayed a wider distribution in the novel tank during the first 3-min of test than black and transparent-confined groups. These 3D reconstructions of behavior have several important advantages over 2D traces because they provide a more “realistic” representation of the fish swimming activity including their lateral movements. A recent study provided a detailed evaluation of three-dimensional neurophenotyping of adult zebrafish behavior [44]. The authors demonstrated that the temporal reconstructions may significantly differ after pharmacological treatments, which allowed the organization of distinct behavioral clusters. This analysis of the swimming pattern has already been applied to create accurate predictive models of medaka fish movement based on high-density trajectory data sets [45], [46]. In addition to the 3D data, we addressed for the first time a new insight of analysis provided by occupancy charts, taking into account not only the distribution, but also the time spent by the fish in each part of the apparatus across time. Thus, the association of both methodologies is a powerful tool which helps to characterize the exploratory profile of zebrafish after environmental manipulations into quantitative models.

Using descriptive ethological diagrams, based on mean frequency, duration and latency of every behavioral pattern, we provided an overview of the spontaneous behavioral patterns displayed by the experimental groups in the open tank. The substantial differences detected in the first 3-min vs. last 3-min of test reflect that the intra-session habituation response to the novel tank involves changes in these behaviors over the course of the test (**see video S1**). The similarities in the ethograms detected for both black and transparent-confined group strongly suggest that it truly is the confinement to white that is affecting the zebrafish behavior away from the baseline.

### Perspectives of the ethological analysis of zebrafish behavior

In conclusion, this study provided detailed approaches to evaluate the spatio-temporal swimming activity and homebase formation of zebrafish during their intra-session habituation period to the novel tank test after a forced exposure to black, white, and transparent cylinders. Since naturalistic approaches may have an important place in research to better understand the biological mechanisms of the behavioral responses in vertebrates [47], the current report supports the idea that zebrafish is undoubtedly a potential animal model for translational research. It must be emphasized that future studies using different protocols could be relevant to further elucidate underlying factors that contribute to the behavioral repertoire observed. One might access the effect promoted by a large spectrum of drugs in the short-period confinement and further subject the animals to the novel tank test. However, researchers are cautioned, at this time, to interpret these data carefully, since the exact significance of the behaviors is not fully understood and little empirical evidence is available to support the validity of the behavioral measures in the open tank [28]–[31]. Furthermore, the current report presented a new analysis of behavioral data by occupancy plots, a quantitative approach for determining the fish exploratory profile, and a detailed ethological analysis in the novel tank. These data help to clarify the ethological network and also bring new insights regarding the validation of spontaneous exploration models. Consequently, the analysis of the overall structure of behavior across time in the open tank task suggests that this paradigm can also be a valuable tool to analyze zebrafish behavioral responses after distinct environmental manipulations.

## Supporting Information

Figure S1 **Spatio-temporal analysis of the exploratory profile of dark, bright, and transparent groups.** (A) Ratio between the total transitions per sections and the number of entries in the respective area during each minute of the trial. * Significant difference between black/transparent and white cylinder-confined groups. Distinct letters mean statistically significant differences within groups (two-way ANOVA followed by Bonferroni's test as post hoc, *p*≤0.05). (B) Representative diagrams demonstrating the transitions per minute estimated by the ratio analysis. The proportion of exploratory activity for each area (bottom, middle, top) and section (1–15) during the novel tank test (6 min) was shown for animals previously confined into dark, bright, and transparent environments.
(TIFF)

Video S1 **Basic behaviors of zebrafish during the intra-session habituation period to the open tank paradigm.** The video describes the protocol of the short-period confinement into dark, bright, and transparent environments (10-min period into the respective cylinder) and demonstrates the spatio-temporal behavior of the experimental groups (dark/transparent vs. bright) in the novel tank diving test. Representative movies of the first 3-min vs. last 3-min for dark/transparent and bright groups were shown (note that the exploratory profile and homebase formation are different between the groups).
(AVI)
