# Supplementary figures and images for: Differences in Spatio-Temporal Behavior of Zebrafish in the Open Tank Paradigm after a Short-Period Confinement into Dark and Bright Environments
Source: PLoS One. 2011 May 2;6(5):e19397. doi: 10.1371/journal.pone.0019397 (PMC3085514; doi:10.1371/journal.pone.0019397)

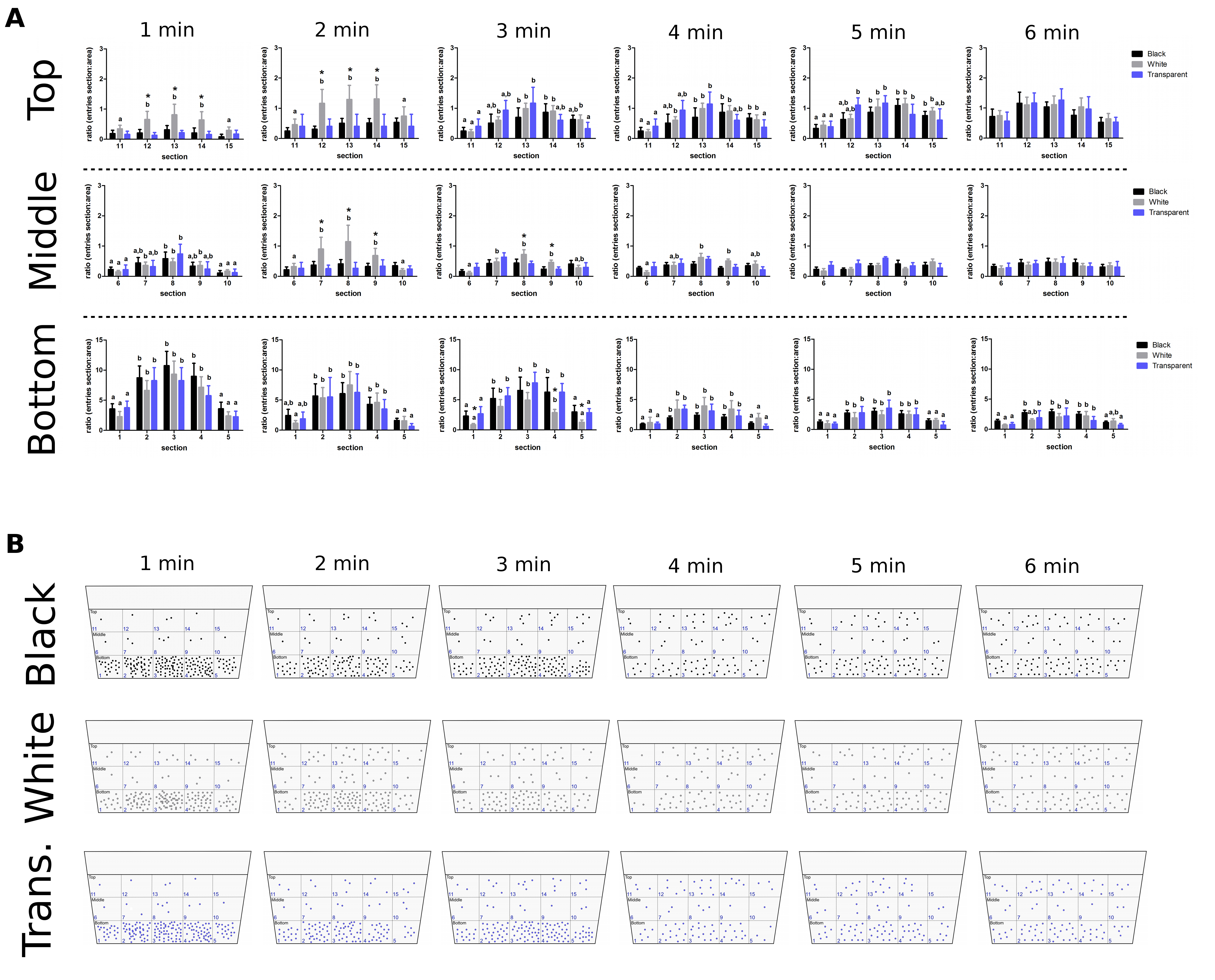

Supplement: Figure S1 — Spatio-temporal analysis of the exploratory profile of dark, bright, and transparent groups. (A) Ratio between the total transitions per sections and the number of entries in the respective area during each minute of the trial. * Significant difference between black/transparent and white cylinder-confined groups. Distinct letters mean statistically significant differences within groups (two-way ANOVA followed by Bonferroni's test as post hoc, p≤0.05). (B) Representative diagrams demonstrating the transitions per minute estimated by the ratio analysis. The proportion of exploratory activity for each area (bottom, middle, top) and section (1–15) during the novel tank test (6 min) was shown for animals previously confined into dark, bright, and transparent environments. (TIFF) [file pone.0019397.s001.tiff]
